# Dynamic augmentation of lip levator function via depressor anguli oris transfer for post-facial paralysis synkinesis

**DOI:** 10.1016/j.jpra.2026.03.044

**Published:** 2026-04-03

**Authors:** Hisashi Sakuma, Eri Matoba, Takako Fujii, Yuma Togashi

**Affiliations:** a Department of Plastic and Reconstructive Surgery, Ichikawa General Hospital, International University of Health and Welfare, Ichikawa, Japan; b Department of Plastic and Reconstructive Surgery, Keio University School of Medicine, Tokyo, Japan

**Keywords:** Facial paralysis, Depressor anguli oris, Synkinesis, Muscle transfer

## Abstract

Post-facial paralysis synkinesis (PFPS) is characterized by the restriction of natural smile due to antagonism between the lip levator muscles and depressor anguli oris (DAO) in the perioral area. Although treatment is often aimed at reducing the DAO alone, which pathologically co-contracts when the mouth corners are elevated, we report a case in which the DAO was turned over and shifted cephalad, thereby alleviating synkinesis and augmenting lip levator function. In a 64-year-old female patient with PFPS due to Hunt syndrome, the DAO flap was elevated from the mandibular origin while preserving the nerve and vascular supply and then inverted cranially to enhance the levator function of the oral commissure. Twelve months after surgery, perioral synkinesis was alleviated, and excursion of the mouth corners and dental show during voluntary and spontaneous smiling were significantly enhanced. This surgical procedure may provide a novel method that can simultaneously improve the dynamic lip levator function and reduce perioral synkinesis in patients with PFPS.

**Lay summary:** This report describes a novel method that can simultaneously improve the dynamic function of the oral commissure and reduce perioral synkinesis during post-facial paralysis synkinesis.

Post-facial paralysis synkinesis (PFPS), a sequela of peripheral facial paralysis, is an intractable condition characterized by facial muscle weakness, synkinesis, hypertonicity, and facial contracture resulting from incomplete recovery.^1^ In particular, co-contraction of the depressor anguli oris (DAO), an antagonist of the lip levator muscles, causes the oral commissure to freeze, making it difficult to smile naturally. This often leads to psychological stress and a decline in quality of life.^1^

Targeted facial neuromuscular therapy and chemodenervation are widely used in the initial management of PFPS. Recently, selective neurectomy or myectomy of the DAO has been performed to weaken the DAO^1^, ^2^, ^3^; however, as there is often a pre-existing weakening of the lip levator muscles, the levator function of the oral commissure is limited.

We devised a novel surgical procedure to alleviate perioral synkinesis and improve the lip levator function via DAO transfer.

## Case report

**Figure 1:**
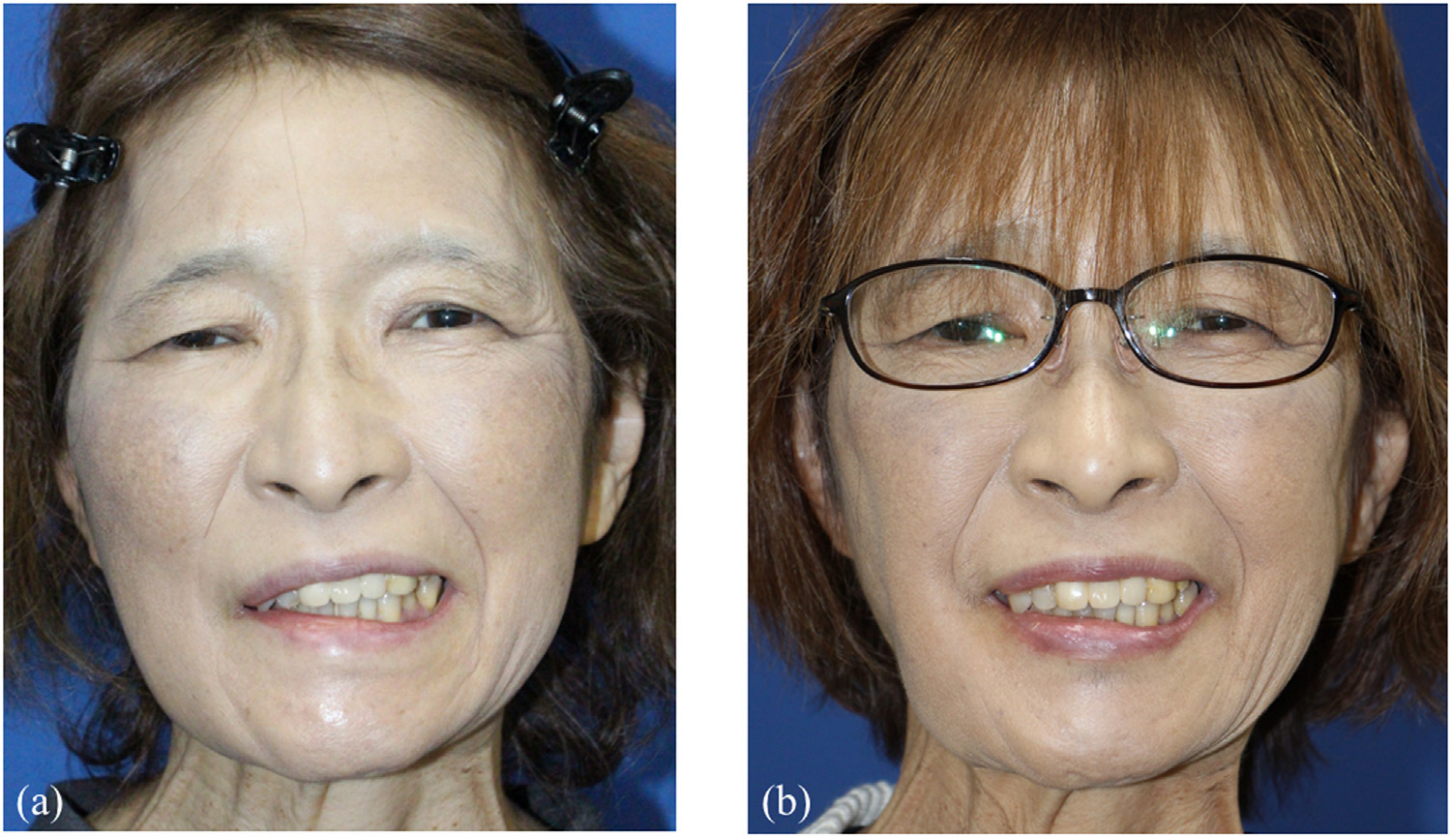
(a) Preoperative view during voluntary smiling. (b) 12 month-postoperative view during voluntary smiling.

**Figure 2:**
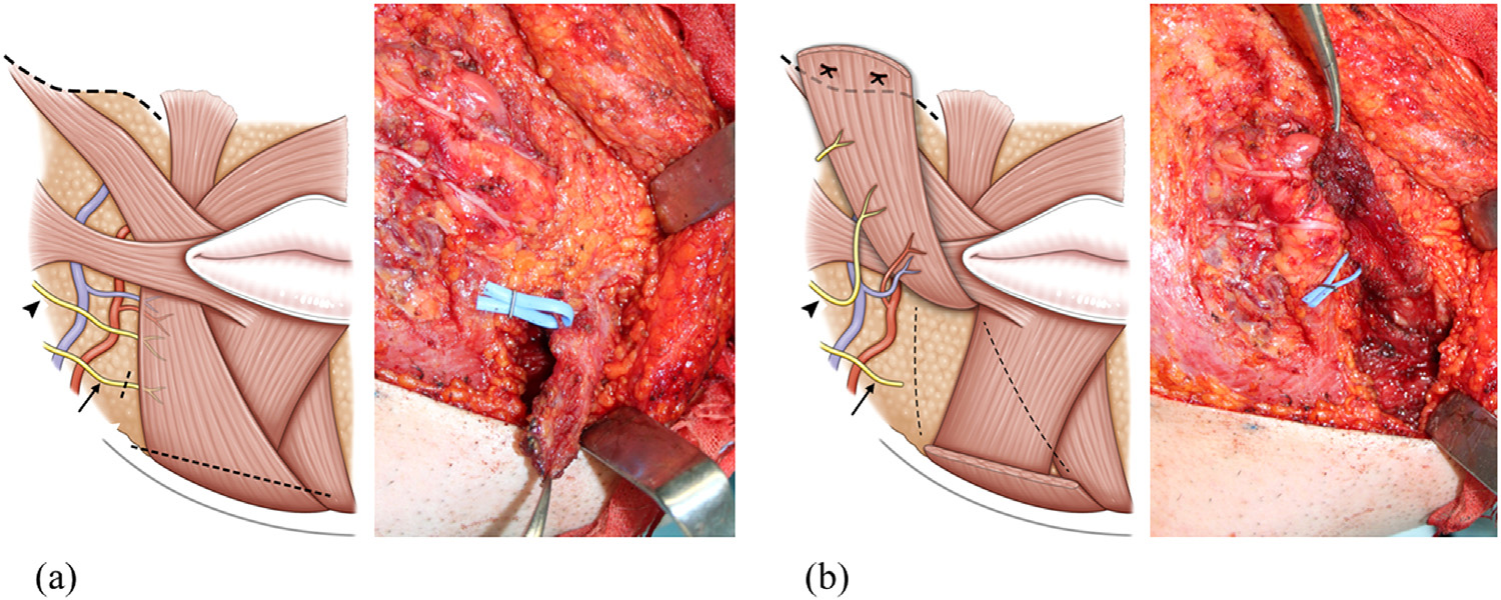
Intraoperative photos and schematic illustrations. (a) After severing the mandibular marginal branch of facial nerve (arrow) into the depressor anguli oris (DAO), the DAO flap is elevated from the mandibular edge to the modiolus, preserving the underlying depressor labii inferioris (DLI), lower buccal branch of facial nerve (arrowhead), and nutrient vessels of the DAO. (b) The elevated muscle flap is then flipped cranially and sutured to the fascia around of the zygomatic body.

A 64-year-old woman experienced right facial paralysis due to Hunt syndrome for 2 years. In addition to the oral-ocular synkinesis associated with lip movements, co-contraction of the DAO, an antagonist of the lip levator muscles, restricts excursion of the oral commissure, resulting in resting and dynamic asymmetry of mouth corners (Figure 1a). To improve these symptoms, we performed selective midfacial neurectomy and augmented lip levator function via DAO transfer. The DAO is 2.5 cm wide at the origin of the mandible and 4 cm from the origin to the insertion of the modiolus. It is nourished by a branch of the facial artery and vein on the cranial side and is innervated by the lower buccal and mandibular marginal branches. Electrical stimulation of the lower buccal branch of facial nerve demonstrated sufficient muscle contraction. After severing the mandibular marginal branch into the DAO, the flap was elevated from the mandibular edge to the modiolus, preserving the lower buccal branch of facial nerve and nutrient vessels of the DAO. The elevated muscle flap was flipped cranially and sutured to the fascia around the zygomatic body (Figure 2).

Twelve months post-surgery, oral-ocular synkinesis was alleviated, and excursion of the oral commissure and dental show during voluntary and spontaneous smile were significantly improved (Figure 1b, Video 1).

## Discussion

Synkinesis, a PFPS symptom, is thought to be caused by misdirection due to aberrant nerve regeneration. It has been theorized that the DAO synkinesis may antagonize oral commissure excursion and that the oral commissure of the affected side is pulled down, impairing the ability to smile.^1^ Therefore, surgical approaches for the DAO, such as selective neurectomy and myectomy, have focused on this as a treatment option.^1^, ^2^, ^3^ In particular, selective myectomy of the DAO led to a significant increase in the oral commissure excursion, smile angle, and dental shows.^2^ Although myectomy of the DAO is effective when there is no inherent weakness of the lip levator muscles, if simply reducing the activity of the smile antagonist muscle is predicted as insufficient to improve smile symmetry, gracilis transfer is necessary for augmentation of the lip levator muscles.

The DAO exhibits a triangular shape, with its origin on the inferior mandibular body and the parasymphysis constituting its base, with its insertion into the modiolus constituting the apex of the triangle. The DAO and DLI receive a segmental blood supply from facial artery branches, entering the full length of the muscles from their lateral border. The DAO receives innervation from the lower buccal branch at the intersection of the upper and middle thirds of the muscle and the marginal mandibular facial nerve branches at the caudal two-thirds of the muscle.^4^

Sarici et al. reported dynamic reconstruction of the orbicularis oris defect of the lower lip using transposition of a DAO flap.^5^ They prepared the muscle as a separate muscle flap and sutured it to the remaining orbicularis oris to achieve muscle continuity and oral competence. The postoperative 1st year electromyography demonstrated decreased intact muscle activity, which was within normal limits. Halani et al. reported transfer of the DAO to the DLI for facial symmetry in synkinetic facial paralysis.^3^ However, this resulted in a lack of a significant increase in excursion and worsened lower lip height deviation.

This surgical procedure is useful, as it allows for easy and safe DAO transfer while preserving the neurovascular pedicle. This procedure that rotates the muscle by severing only its origin, also helps prevent distortion of the modiolus and mouth corners. Furthermore, it can simultaneously improve perioral synkinesis and compensate for lip levator muscle weakness, which are PFPS symptoms, by adding the DAO to the same vector that co-contracts owing to aberrant nerve regeneration.

The limitation of this surgical procedure is that modiolus insertion of the DAO is slightly closer to the lower lip. Therefore, in order to obtain further elevation of the upper lip, improvements such as re-fixing the DAO insertion point closer to the upper lip may be necessary.

## Funding

This study was supported by JSPS KAKENHI (Grant Number JP 25K12919).

## Patient consent

A patient provided written informed consent for the publication and the use of her images.

## Declarations of competing interest

None of the authors has any financial interests to declare in relation to the contents of this article.

## Appendix Supplementary materials

Preoperative and 12-month-postoperative video during voluntary and spontaneous smiling. Image, video 1 dummy alt text
